# Lumbar safety triangle: comparative study of coronal and coronal oblique planes in 3.0-T magnetic resonance imaging

**DOI:** 10.1590/0100-3984.2023.0022

**Published:** 2023

**Authors:** Fernando Augusto Dannebrock, Erasmo de Abreu Zardo, Marcus Sofia Ziegler, Emiliano Vialle, Ricardo Bernardi Soder, Carla Helena Augustin Schwanke

**Affiliations:** 1 Pontifícia Universidade Católica do Rio Grande do Sul (PUCRS), Porto Alegre, RS, Brazil.; 2 Instituto Gaúcho de Cirurgia da Coluna Vertebral, Porto Alegre, RS, Brazil.; 3 Hospital Universitário Cajuru, Curitiba, PR, Brazil.

**Keywords:** Spine, Spinal ganglia, Magnetic resonance imaging, Minimally invasive surgical procedures, Spinal nerve roots, Coluna vertebral, Gânglios espinais, Ressonância magnética, Procedimentos cirúrgicos minimamente invasivos, Raízes nervosas espinais

## Abstract

**Objective:**

To compare the measurements of the lumbar safety triangle (Kambin’s triangle) and the invasion of the dorsal root ganglion in the triangle in coronal and coronal oblique planes.

**Materials and Methods:**

A cross-sectional study, in which 210 3.0-T magnetic resonance images of L2-L5 were analyzed in coronal and coronal oblique planes. Exams with lumbar spine anomalies were excluded. Demographic (sex and age) and radiological variables were recorded by a single evaluator.

**Results:**

Most sample was female (57.1%), mean age 45.5 ± 13.3 (18–98 years). The measurements average, as well as the areas, gradually increased from L2 to L5. The dorsal root ganglion invaded the triangle in all images. The safety triangle average area was smaller in the coronal oblique plane than in the coronal plane. Of the seven dimensions of safety triangle obtained for each level of the lumbar spine, six were significantly smaller in the coronal oblique plane than in the coronal plane. The only dimension that showed no difference was the smallest ganglion dimension.

**Conclusion:**

The dimensions and areas investigated were smaller in coronal oblique plane, especially the area (difference > 1 mm). The analysis of the triangular zone in this plane becomes important in the preoperative assessment of minimally invasive procedures.

## Abstract (pt)

**Objetivo:**

Comparar as medidas do triângulo de segurança lombar (triângulo de Kambin) e invasão do gânglio da raiz dorsal no triângulo nas incidências coronal e coronal oblíqua.

**Materiais e Métodos:**

Estudo transversal, em que foram analisadas 210 imagens de ressonância magnética 3.0-T de L2-L5 nos planos coronal e coronal oblíquo. Foram excluídos exames com anomalias da coluna lombar. Variáveis demográficas (sexo e idade) e radiológicas foram registradas por um único avaliador.

**Resultados:**

A maioria da amostra era do sexo feminino (57,1%), com idade média de 45,5 ± 13,3 (18–98 anos). A média das medidas, assim como as áreas, aumentaram gradativamente de L2 a L5. O gânglio da raiz dorsal invadiu o triângulo em todas as imagens. A área média do triângulo de segurança foi menor na incidência coronal oblíqua do que na incidência coronal. Das sete dimensões do triângulo de segurança obtidas para cada nível da coluna lombar, seis foram significativamente menores no plano coronal oblíquo do que no plano coronal. Única dimensão que não apresentou diferença foi a menor dimensão do gânglio.

**Conclusão:**

As dimensões e áreas investigadas foram menores na incidência coronal oblíqua, especialmente a área (diferença > 1 mm). A análise da zona triangular nesta incidência torna-se importante na avaliação pré-operatória de procedimentos minimamente invasivos.

## INTRODUCTION

Minimally invasive surgical techniques for procedures involving the lumbar region of the spine gained popularity from the beginning of the 21st century. For the performance of lumbar posterolateral percutaneous procedures, accurate knowledge of the surgical anatomy is of fundamental importance for safely accessing the intervertebral disc^(1,2,3,4)^.

Parvis Kambin, in 1983, described a corridor of safe access to the intervertebral disc, known as Kambin’s triangle, the lumbar safety triangle, or the triangular safety zone^(5)^. This zone is described as having the dura mater as its medial boundary, the upper vertebral plateau as its lower boundary, and the nerve root as its hypotenuse^(6)^. Initial descriptions of the lumbar safety triangle were based on anatomical studies of cadavers. However, cadavers undergo structural changes over time, mainly due to the decrease in tension within the tissues^(7)^. After new imaging methods, especially magnetic resonance imaging (MRI), came into use, the scientific literature began to explore the assessment of the triangle by using these methods. Because the bone structure and the surgical trajectory are three-dimensional, the modality of choice is 3.0-T MRI, which generates images in high definition, translating to greater spatial resolution and thus increasing the safety of the procedures^(8,9,10,11)^.

Although there have been many studies of the lumber safety triangle in cadavers, only a few have involved the use of MRI^(12,13,14,15,16)^. To our knowledge, there have been no studies analyzing the dimensions of the dorsal root ganglion and its relationship with the lumbar safety triangle in different planes. The description of that structure is important in minimally invasive procedures because it is a grouping of sensory fibers that is also related to the mechanism of radiculopathy^(17,18,19,20)^. In a previous study, Dannebrock et al.^(12)^ analyzed the dimensions of the lumbar safety triangle in the coronal plane, reporting that the dorsal root ganglion invaded the boundaries of the triangle in all of the images evaluated.

The lumbar safety triangle has been analyzed in different MRI planes, especially the coronal and sagittal planes^(16,21)^. It is also important to analyze the coronal oblique plane, given that the insertion of the working cannulas during surgery occurs in that plane^(22)^. Analysis in the coronal oblique plane potentially makes the preoperative assessment more reliable and helps reduce the risk of intraoperative complications. Therefore, this study aims to compare the area and measurements of the lumbar safety triangle obtained in the coronal and coronal oblique planes at the L2–L3, L3–L4, and L4–L5 levels in patients undergoing 3.0-T MRI, as well as to determine whether there are age- or sex-related differences.

## MATERIALS AND METHODS

This was a retrospective cross-sectional study. The research project was approved by the Research Ethics Committee of the Pontifícia Universidade Católica do Rio Grande do Sul (PUCRS)—Reference No 3.902.008, CAAE No. 24551019.0.0000.5336. All procedures were performed in accordance with the relevant guidelines and regulations. Because of the retrospective nature of the study, the requirement for informed consent was waived. However, the researchers signed a confidentiality agreement to ensure the anonymity of the data obtained. Thus, all of the researchers involved in the study gave written informed consent in accordance with the guidelines approved by the PUCRS Research Ethics Committee.

We selected all 3.0-T MRI images of the lumbosacral spine of patients ≥ 18 years of age who underwent the examination at the Instituto do Cérebro do Rio Grande do Sul (InsCer) between December of 2017 and December of 2020. Images that presented disease (including disc herniation, foraminal stenosis, scoliosis, and others that would change the shape of the lumbar safety triangle) were excluded, as were those showing evidence of previous surgery of the lumbosacral spine (described in the MRI report or identified in the image analysis). If more than one MRI imaging study was performed during the study period, only the first was considered.

### Sample size and minimal clinically important difference

The minimal clinically important difference was determined by using the distribution method with the standard error of the mean (SEM) formula, established as the divergence between methods, deemed greater than the estimated value of 1 SEM according to the available literature. To compute the mean values of the lumbar safety triangle area within coronal planes at the L2–L3, L3–L4, and L4–L5 levels on 3.0-T MRI scans, we relied on the data presented by Dannebrock et al.^(12)^. The present study comprised 101 patients who underwent 3.0-T MRI, and we leveraged their data to determine the SEM for each level: L2–L3 = 2.46; L3–L4 = 4.18; and L4–L5 = 3.67.

We performed an *a priori* analysis and established that a sample size of 90 patients would guarantee a statistical power of 80% with a significance level of 5%. On the basis of Cohen’s effect size statistic^(23)^, this sample size was considered sufficient for identifying at least a moderate effect size (d = 0.3) between the coronal and coronal oblique planes. The mean effect size was determined as the minimal clinically important difference for the mean divergence between the coronal plane and the coronal oblique plane in terms of the area of the lumbar safety triangle.

## 3.0-T MRI

All of the patients underwent MRI of the lumbosacral spine, in the coronal and coronal oblique planes, in a 3.0-T scanner (Signa HDXT; GE HealthCare, Chicago, IL, USA), with a spine coil. With the patient in the supine position, accelerated T2-weighted fast spin-echo sequences were performed in the coronal and coronal oblique planes with the following parameters: field of view, 32 cm; slice thickness, 2 mm; interslice gap, 0.2 mm; matrix, 448 × 320; number of slices, 22; repetition time/echo time, 3,700/80 ms. Images were collected with maximum intensity projection reconstruction, with an increment of 0.5 mm, thickness of 5 mm, and inclination of 30° in the coronal oblique plane.

### Research variables

**Figure 1:**
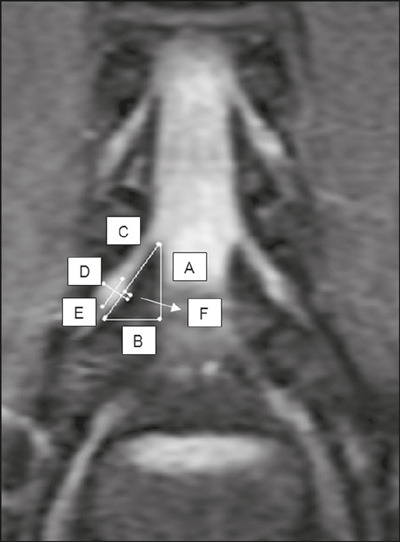
Coronal 3.0-T MRI slice acquired at the L4–L5 level to the right of the lumbar spine, showing the respective boundaries of the lumbar safety triangle and its relationship with the dorsal root ganglion. Height (A), base (B), hypotenuse (C), largest dimension of the ganglion (D), smallest dimension of the ganglion (E), and degree of ganglion invasion into the triangle (F).

**Figure 2:**
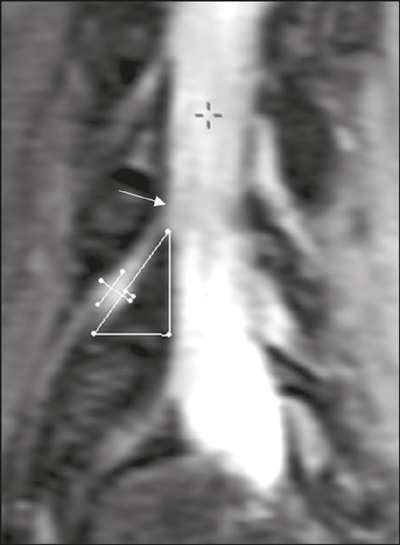
Anatomical aspect in the coronal oblique plane at the L4-L5 level to the right of the lumbar spine, obtained by 3.0-T MRI, showing with the respective boundaries of the lumbar safety triangle and its relationship with the dorsal root ganglion. The height, base, hypotenuse, dorsal root ganglion boundaries (largest and smallest dimensions), and degree of ganglion invasion into the triangle are as illustrated in Figure 1.

The ages (to determine the age group) and sexes of the patients were recorded. On MRI, the height, base, hypotenuse, and area of the lumbar safety triangle were determined at the L2–L3, L3–L4, and L4–L5 levels on the right side, as described in Table 1, which also shows how the dimensions of the dorsal root ganglion and its location relative to the triangle were determined. The L1–L2 and L5–S1 segments were not studied because of the technical limitations of the images obtained (full slices were not available). Measurements were performed in the coronal and coronal oblique planes (Figures 1 and 2), using a digital ruler available in the image interpretation software developed at the InsCer (Arya). All measurements were obtained by a researcher with a background in orthopedics/traumatology and specialization in spinal surgery, who was trained by a radiologist with experience in musculoskeletal analysis.

**Table 1:** Description of how the radiological variables were obtained.

Boundaries of the lumbar safety triangle	Height or medial boundary (mm): defined as the lateral edge of the dura mater, being measured from the upper border of the lower vertebra in the caudal portion to the cranial border that corresponds to the upper edge of the nerve root.
	Hypotenuse (mm): corresponds to the spinal nerve (lumbar root), being measured from its beginning at the lateral edge of the dura mater to its lower border, which corresponds to the upper plateau of the lower vertebra.
	Base (mm): measured from the lateral edge of the dura mater to the lateral border of the corresponding lumbar nerve root, with the upper vertebral plateau of the lower vertebra as the lower border.
Area of the lumbar safety triangle	Determined by using free software with a specific tool for calculating the area (in mm^3^) from MRI slices of the lumbosacral spine in the coronal and coronal oblique planes.
Dorsal root ganglion dimensions and location in relation to the lumbar safety triangle	Measurement of the largest and smallest dimensions (in mm) of the ganglion, determination of whether or not it invaded the lumbar safety triangle, and evaluation of the degree of ganglion invasion (in mm) into the triangle.

### Statistical analysis

Data were stored in an Excel spreadsheet and analyzed with the IBM SPSS Statistics software package, version 21.0 (IBM Corp.; Armonk, NY, USA). The normality of the continuous data distribution was determined by using the Kolmogorov-Smirnov test, and all variables presented normal distribution. Numerical data are expressed as mean and standard deviation, whereas categorical data are expressed as absolute and relative frequencies. Student’s t-test, paired t-test, and analysis of variance were used in order to compare continuous measurements. Values of *p* < 0.05 were considered significant.

## RESULTS

**Figure 3:**
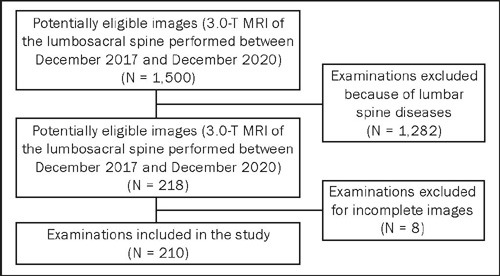
Flow chart of the image selection process.

We analyzed a total of 210 3.0-T MRI images, comprising 1,260 lumbar safety triangles (Figure 3). The mean age of the patients was 45.5 ± 13.3 years (range, 18–98 years). Of the 101 patients evaluated, 56 (55.2%) were between 40 and 65 years of age and 58 (57.1%) were female.

Table 2 shows the measures of the lumbar safety triangle, in the coronal plane on the right side, by sex and age group. At the L2–L3 level, the means of five of the seven measures were significantly higher among the men than among the women, including the height, hypotenuse, and area of the lumbar safety triangle (*p* = 0.017, *p* = 0.009, and *p* = 0.018, respectively), as well as the largest and smallest dimensions of the dorsal root ganglion (*p* = 0.002 and *p* < 0.001, respectively). The measures of the base of the lumbar safety triangle and the degree of ganglion invasion into the triangle showed no differences between the sexes (*p* = 0.069 and *p* = 0.190, respectively). At the L3–L4 level, all of the measures were significantly higher among the men: height of the triangle (*p* = 0.015); base of the triangle (*p* = 0.028); hypotenuse of the triangle (*p* = 0.007); area of the triangle *(p* = 0.009); largest dimension of the ganglion (*p* < 0.001); smallest dimension of the ganglion (*p* = 0.001); and degree of ganglion invasion into the triangle (*p* = 0.040). At the L4–L5 level, all of the measures were also significantly higher among the men: height of the triangle (*p* < 0.001); base of the triangle (*p* = 0.001); hypotenuse of the triangle (*p* < 0.001); area of the triangle (*p* < 0.001); largest dimension of the ganglion (*p* < 0.001); smallest dimension of the ganglion (*p* = 0.003); and degree of ganglion invasion into the triangle (*p* = 0.010). The analysis by age group showed that, in the coronal plane, just two of the seven measures were statistically different at the L2–L3 level: the measures of the base and area of the lumbar safety triangle were significantly lower in the 18- to 39-year age group than in the 40- to 65-year age group (*p* = 0.0016 and *p* = 0.038, respectively). At the L3–L4 and L4–L5 levels, there were no statistically significant differences among the age groups (*p* > 0.005 for all parameters).

**Table 2:** Parameters of the lumbar safety triangle and dorsal root ganglion, in the coronal plane on the right side, by sex and age group, at the different levels of the lumbar spine.

Parameter	Total sample (N = 210) Mean ± SD (range)	Sex			Age group			
		Female (n = 120) Mean ± SD	Male (n = 90) Mean ± SD	p*	18–39 years (n = 75) Mean ± SD	40–65 years (n = 116) Mean ± SD	66–98 years (n = 19) Mean ± SD	*p*†
At L2–L3								
Lumbar safety triangle								
Height, mm	14.9 ± 2.7 (8.6–22.6)	14.5 ± 2.7	15.4 ± 2.7	**0.017**	14.5 ± 2.8	15.2 ± 2.7	14.7 ± 2.1	0.254
Base, mm	11.8 ± 2.6 (6.8–19.3)	11.5 ± 2.4	12.2 ± 2.8	0.069	11.1 ± 2.5^a^	12.2 ± 2.6^b^	12.0 ± 2.3^a^, ^b^	**0.016**
Hypotenuse, mm	15.5 ± 3.3 (7.3–23.7)	15.0 ± 3.2	16.2 ± 3.3	**0.009**	15.0 ± 3.2	15.8 ± 3.4	15.7 ± 2.5	0.233
Area, mm^3^	90.0 ± 32.7 (37.8–191.0)	85.3 ± 29.3	96.4 ± 35.9	**0.018**	82.7 ± 31.5^a^	95.0 ± 33.9^b^	89.1 ± 25.0^a^, ^b^	**0.038**
Dorsal root ganglion								
Largest dimension, mm	6.1 ± 1.0 (3.3–9.2)	5.9 ± 0.9	6.4 ± 1.1	**0.002**	6.1 ± 1.0	6.1 ± 1.0	6.2 ± 1.0	0.888
Smallest dimension, mm	5.0 ± 0.8 (3.1–7.0)	4.8 ± 0.7	5.3 ± 0.9	**< 0.001**	5.1 ± 0.8	5.0 ± 0.8	4.9 ± 0.6	0.600
Invasion into the triangle, mm	1.1 ± 0.2 (0.6–1.5)	1.0 ± 0.2	1.1 ± 0.2	0.190	1.1 ± 0.2	1.1 ± 0.2	1.1 ± 0.2	0.886
At L3–L4								
Lumbar safety triangle								
Height, mm	15.7 ± 2.6 (9.9–25.2)	15.3 ± 2.6	16.2 ± 2.5	**0.015**	15.3 ± 2.8	15.9 ± 2.5	15.4 ± 1.8	0.286
Base, mm	12.2 ± 2.5 (6.9–19.5)	11.8 ± 2.3	12.6 ± 2.7	**0.028**	11.6 ± 2.5	12.5 ± 2.6	12.1 ± 1.9	0.069
Hypotenuse, mm	16.2 ± 3.1 (8.6–23.8)	15.7 ± 2.9	16.9 ± 3.2	**0.007**	15.5 ± 3.1	16.6 ± 3.2	16.5 ± 2.3	0.060
Area, mm^3^	97.4 ± 32.8 (34.1–204.7)	92.3 ± 30.0	104.2 ± 35.7	**0.009**	91.4 ± 33.4	101.8 ± 33.6	94.2 ± 21.2	0.093
Dorsal root ganglion								
Largest dimension, mm	6.4 ± 0.9 (4.2–9.6)	6.2 ± 0.8	6.7 ± 1.0	**< 0.001**	6.4 ± 0.9	6.5 ± 0.9	6.4 ± 1.0	0.983
Smallest dimension, mm	5.2 ± 0.8 (3.2–7.3)	5.1 ± 0.7	5.4 ± 0.9	**0.001**	5.3 ± 0.8	5.2 ± 0.8	5.1 ± 0.7	0.714
Invasion into the triangle, mm	1.1 ± 0.2 (0.7–1.6)	1.1 ± 0.2	1.2 ± 0.2	**0.040**	1.1 ± 0.2	1.1 ± 0.2	1.2 ± 0.1	0.736
At L4–L5								
Lumbar safety triangle								
Height, mm	16.4 ± 2.6 (9.4–28.4)	15.7 ± 2.3	17.3 ± 2.7	**< 0.001**	16.3 ± 3.0	15.5 ± 2.5	16.1 ± 1.7	0.670
Base, mm	12.9 ± 2.3 (6.9–19.7)	12.4 ± 2.1	13.5 ± 2.4	**0.001**	12.5 ± 2.3	13.2 ± 2.3	12.6 ± 2.0	0.155
Hypotenuse, mm	17.3 ± 3.0 (10.5–26.4)	16.5 ± 2.8	18.3 ± 3.0	**< 0.001**	17.0 ± 3.2	17.4 ± 2.9	17.2 ± 2.3	0.638
Area, mm^3^	107.5 ± 32.8 (45.5–242.8)	99.1 ± 27.8	118.6 ± 35.7	**< 0.001**	104.1 ± 35.6	110.5 ± 32.2	102.1 ± 22.7	0.318
Dorsal root ganglion								
Largest dimension, mm	6.7 ± 1.0 (4.2–10.4)	6.4 ± 0.8	7.0 ± 1.0	**< 0.001**	6.7 ± 0.9	6.7 ± 1.0	6.6 ± 1.2	0.952
Smallest dimension, mm	5.3 ± 0.8 (2.9–8.4)	5.2 ± 0.7	5.5 ± 0.9	**0.003**	5.4 ± 0.8	5.3 ± 0.8	5.2 ± 0.2	0.414
Invasion into the triangle, mm	1.2 ± 0.2 (0.6–1.8)	1.2 ± 0.2	1.3 ± 0.2	**0.010**	1.2 ± 0.2	1.2 ± 0.2	1.2 ± 0.2	0.844

*Student’s t-test. †Analysis of variance with Bonferroni post-hoc analysis (different superscript letters in the same row indicate a statistically significant difference).

Table 3 shows the seven measures obtained at each level of the lumbar spine, in the coronal oblique plane on the right side, by sex and age group. At the L2–L3 level, the means of five measures were significantly higher in the men than in the women, including the height, hypotenuse, and area of the lumbar safety triangle (*p* = 0.030, *p* = 0.022, and *p* = 0.024, respectively), as well as the largest and smallest dimensions of the dorsal root ganglion (*p* = 0.001 and *p* < 0.001, respectively). The measures of the triangle base and the degree of ganglion invasion into the triangle showed no statistically significant difference between the sexes (*p* = 0.063 and *p* = 0.245, respectively). At the L3–L4 and L4–L5 level, respectively, most of the measures were significantly higher in men—triangle height (*p* = 0.025 and *p* < 0.001); triangle base (*p* = 0.011 and *p* = 0.001); triangle hypotenuse (*p* = 0.010 and *p* < 0.001); triangle area (*p* = 0.007 and *p* < 0.001); largest dimension of the ganglion (*p* = 0.001 and *p* < 0.001); and smallest dimension of the ganglion (*p* < 0.001 for both)—the sole exception being the degree of ganglion invasion into the triangle (*p* = 0.100 and *p* = 0.155). The analysis by age group showed that, in the coronal oblique plane, the base and area of the triangle at the L2–L3 level were significantly smaller in the 18- to 39-year age group than in the 40- to 65-year age group (*p* = 0.014 and *p* = 0.029, respectively). At the L3–L4 level, only the hypotenuse was significantly different, being smaller in the 18- to 39-year age group than in the 40- to 65-year age group (*p* = 0.045). At the L4–L5 level, none of the measures showed a statistically significant difference among the age groups (*p* > 0.005 for all).

**Table 3:** Parameters of the lumbar safety triangle and dorsal root ganglion, in the coronal oblique plane on the right side, by sex and age group, at the different levels of the lumbar spine.

Parameter	Total sample (N = 210) Mean ± SD (range)	Sex			Age group			
		Female (n = 120) Mean ± SD	Male (n = 90) Mean ± SD	p*	18–39 years (n = 75) Mean ± SD	40–65 years (n = 116) Mean ± SD	66–98 years (n = 19) Mean ± SD	*p*†
At L2–L3								
Lumbar safety triangle								
Height, mm	14.7 ± 2.7 (8.7–22.2)	14.4 ± 2.7	15.2 ± 2.8	**0.030**	14.3 ± 2.8	15.0 ± 2.8	14.6 ± 2.1	0.188
Base, mm	11.7 ± 2.5 (6.8–19.1)	11.4 ± 2.4	12.1 ± 2.7	0.063	11.4 ± 2.4^a^	12.1 ± 2.6^b^	11.8 ± 2.3^a^, ^b^	**0.014**
Hypotenuse, mm	15.3 ± 3.3 (7.6–23.4)	14.8 ± 3.2	15.9 ± 3.5	**0.022**	14.7 ± 3.3	15.6 ± 3.4	15.5 ± 2.6	0.181
Area, mm^3^	88.5 ± 32.2 (36.9–187.1)	84.1 ± 29.0	94.5 ± 35.3	**0.024**	81.1 ± 31.0^a^	93.6 ± 33.3^b^	86.9 ± 24.4^a^, ^b^	**0.029**
Dorsal root ganglion								
Largest dimension, mm	6.0 ± 1.0 (3.3–9.1)	5.8 ± 0.9	6.3 ± 1.1	**0.001**	6.0 ± 1.1	6.0 ± 1.0	6.0 ± 1.0	0.978
Smallest dimension, mm	5.0 ± 0.7 (3.0–6.80	4.8 ± 06	5.2 ± 0.8	**< 0.001**	5.0 ± 0.8	5.0 ± 0.7	4.9 ± 0.6	0.632
Invasion into the triangle, mm	1.0 ± 0.2 (0.5–1.5)	1.0 ± 0.2	1.0 ± 0.2	0.245	1.0 ± 0.2	1.0 ± 0.2	1.0 ± 0.2	0.931
At L3–L4								
Lumbar safety triangle								
Height, mm	15.4 ± 2.6 (10.0–25.2)	15.1 ± 2.6	15.9 ± 2.6	**0.025**	15.1 ± 2.8	15.7 ± 2.5	15.1 ± 1.9	0.238
Base, mm	12.1 ± 2.4 (7.5–19.2)	11.7 ± 2.2	12.6 ± 2.6	**0.011**	11.6 ± 2.4	12.4 ± 2.5	12.0 ± 1.8	0.086
Hypotenuse, mm	16.0 ± 3.1 (8.1–23.7)	15.5 ± 3.0	16.6 ± 3.2	**0.010**	15.2 ± 3.1^a^	16.4 ± 2.4^b^	16.2 ± 2.4^a^, ^b^	**0.045**
Area, mm^3^	95.5 ± 32.1 (38.5–198.7)	90.3 ± 29.0	102.4 ± 34.8	**0.007**	89.8 ± 32.7	99.7 ± 32.8	91.8 ± 21.1	0.101
Dorsal root ganglion								
Largest dimension, mm	6.3 ± 0.9 (4.0–9.2)	6.1 ± 0.8	6.6 ± 1.0	**0.001**	6.3 ± 0.9	6.3 ± 0.9	6.3 ± 1.0	0.956
Smallest dimension, mm	5.2 ± 0.7 (3.1–7.2)	5.0 ± 0.6	5.5 ± 0.8	**< 0.001**	5.3 ± 0.7	5.2 ± 0.7	5.0 ± 0.7	0.195
Invasion into the triangle, mm	1.1 ± 0.2 (06–1.5)	1.1 ± 0.1	1.1 ± 0.2	0.100	1.1 ± 0.2	1.1 ± 0.2	1.1 ± 0.1	0.942
At L4–L5								
Lumbar safety triangle								
Height, mm	16.1 ± 2.6 (9.2–26.9)	15.5 ± 2.3	17.0 ± 2.8	**< 0.001**	16.0 ± 2.9	16.3 ± 2.5	15.8 ± 1.7	0.531
Base, mm	12.8 ± 2.3 (7.0–19.5)	12.3 ± 2.0	13.4 ± 2.4	**0.001**	12.5 ± 2.3	13.0 ± 2.3	12.5 ± 1.9	0.311
Hypotenuse, mm	16.9 ± 3.0 (10.1–25.5)	16.2 ± 2.7	17.8 ± 3.2	**< 0.001**	16.5 ± 3.2	17.1 ± 3.0	16.8 ± 2.4	0.409
Area, mm^3^	105.0 ± 32.8 (41.8–242.1)	97.0 ± 27.1	115.6 ± 36.6	**< 0.001**	102.2 ± 36.1	107.7 ± 31.9	99.1 ± 22.2	0.377
Dorsal root ganglion								
Largest dimension, mm	6.5 ± 0.9 (4.0–9.8)	6.3 ± 0.8	6.9 ± 1.0	**< 0.001**	6.5 ± 0.9	6.5 ± 0.9	6.5 ± 1.1	0.976
Smallest dimension, mm	5.4 ± 0.8 (3.3–8.1)	5.2 ± 0.7	5.6 ± 0.9	**< 0.001**	5.5 ± 0.7	5.3 ± 0.8	5.1 ± 0.7	0.082
Invasion into the triangle, mm	1.1 ± 0.2 (0.7–1.6)	1.1 ± 0.1	1.2 ± 0.2	0.155	1.1 ± 0.2	1.1 ± 0.2	1.1 ± 0.2	0.998

*Student’s t-test. †Analysis of variance with Bonferroni post-hoc analysis (different superscript letters in the same row indicate a statistically significant difference).

We observed that the mean values for the measures gradually increased from the L2–L3 level to the L4–L5 level, in both planes. We also found that the dorsal root ganglion invaded the lumbar safety triangle in all of the images evaluated.

Table 4 shows the comparison between the right-sided lumbar safety triangle measures obtained in the coronal plane and those obtained in the coronal oblique plane. At the L2–L3 level, six of the seven measures were significantly smaller in the coronal oblique plane than in the coronal plane: triangle height (*p* < 0.001); triangle base (*p* = 0.003); triangle hypotenuse (*p* < 0.001); triangle area (*p* < 0.001); largest dimension of the dorsal root ganglion (*p* < 0.001); and smallest dimension of the ganglion (*p* < 0.001). At the L3–L4 and L4–L5 levels, those same measures were also significantly smaller in the coronal oblique plane: triangle height (*p* < 0.001 for both); triangle base (*p* = 0.050 and *p* = 0.003, respectively); triangle hypotenuse (*p* < 0.001 for both); triangle area (*p* < 0.001 for both); largest dimension of the ganglion (*p* < 0.001 for both); and smallest dimension of the ganglion (*p* < 0.001 for both). The differences between the triangle areas obtained in the coronal oblique plane and those obtained in the coronal plane were greater than 1 mm. The smallest dimension of the ganglion was the only measure that did not show a statistically significant difference at any of the levels (*p* > 0.05 for all).

**Table 4:** Parameters of the lumbar safety triangle and dorsal root ganglion, on the right side, by plane.

Variables (mm)	Plane		p*
	Coronal Mean ± SD	Coronal oblique Mean ± SD	
At L2–L3			
Lumbar safety triangle			
Height, mm	14.9 ± 2.7	14.7 ± 2.7	**< 0.001**
Base, mm	11.8 ± 2.6	11.7 ± 2.5	**0.003**
Hypotenuse, mm	15.5 ± 3.3	15.3 ± 3.3	**< 0.001**
Area, mm^3^	90.0 ± 32.7	88.5 ± 32.2	**< 0.001**
Dorsal root ganglion			
Largest dimension, mm	6.1 ± 1.0	6.0 ± 1.0	**< 0.001**
Smallest dimension, mm	5.0 ± 0.8	5.0 ± 0.7	0.461
Invasion into the triangle, mm	1.1 ± 0.2	1.0 ± 0.2	**< 0.001**
At L3–L4			
Lumbar safety triangle			
Height, mm	15.7 ± 2.6	15.4 ± 2.6	**< 0.001**
Base, mm	12.2 ± 2.5	12.1 ± 2.4	**0.050**
Hypotenuse, mm	16.2 ± 3.1	16.0 ± 3.1	**< 0.001**
Area, mm^3^	97.4 ± 32.8	95.5 ± 32.1	**< 0.001**
Dorsal root ganglion			
Largest dimension, mm	6.4 ± 0.9	6.3 ± 0.9	**< 0.001**
Smallest dimension, mm	5.2 ± 0.8	5.2 ± 0.7	0.751
Invasion into the triangle, mm	1.14 ± 0.17	1.10 ± 0.16	**< 0.001**
At L4–L5			
Lumbar safety triangle			
Height, mm	16.4 ± 2.6	16.1 ± 2.6	**< 0.001**
Base, mm	12.9 ± 2.3	12.8 ± 2.3	**< 0.001**
Hypotenuse, mm	17.3 ± 3.0	16.9 ± 3.0	**< 0.001**
Area, mm^3^	107.5 ± 32.8	105.0 ± 32.8	**< 0.001**
Dorsal root ganglion			
Largest dimension, mm	6.7 ± 1.0	6.5 ± 0.9	**< 0.001**
Smallest dimension, mm	5.3 ± 0.8	5.4 ± 0.8	0.343
Invasion into the triangle, mm	1.2 ± 0.2	1.1 ± 0.2	**< 0.001**

## DISCUSSION

Because few of the studies analyzing the lumbar safety triangle have evaluated images obtained in an oblique plane, we have described and compared the boundaries of this structure in the coronal and coronal oblique planes. The most interesting finding of our study was that there were differences of more than 1 mm between the triangle areas measures in the coronal plane and those measured in the coronal oblique plane, given that such a size difference might be relevant in daily surgical practice.

Despite the relative safety of minimally invasive techniques, they are susceptible to complications. Such complications are usually related to the nerve root, especially the dorsal root ganglion^(24,25)^, and their incidence can be reduced by careful preoperative image analysis^(26)^. The incidence of complications in minimally invasive procedures is approximately 1%^(27)^, the most common complication being dysesthesia, a neurological disorder of the sensory ganglion characterized by weakening of or alteration in the sensitivity of the senses, especially touch. Most cases of postoperative dysesthesia resolve within approximately 60 days with pharmacological treatment^(28)^.

Tumialán et al.^(29)^ provided an overview of the history and controversies surrounding the lumbar safety triangle. The authors stated that the term Kambin’s triangle should be used only in the context of percutaneous access to the disc space for endoscopic procedures in the intact spine and should not be applied in cases of patients undergoing transforaminal lumbar interbody fusion after laminectomy and facetectomy.

In the literature, there is some divergence regarding the true form of the lumbar safety triangle^(30)^. In one anatomical study of cadavers, Ozer et al.^(31)^ described three different variants of the triangle–type 1, no apparent triangle; type 2, a small triangle; and type 3, a normal triangle (Kambin’s original triangle)—with the variant described by Kambin being the least common. However, the authors of that study did not mention the dorsal root ganglion^(31)^. In the present study, which involved the analysis of high-definition images, most patients were found to present the third variant. In an anatomical MRI study, Dannebrock et al.^(12)^ reported similar findings. One possible explanation for these divergent findings would be the fact that the Ozer et al.^(31)^ study was a cadaver study. Although cadavers are frequently used in the study of the lumbar safety triangle, they can suffer tissue retraction and structural alterations of as much as 17% over time^(7)^.

In the present study, the dimensions of the three sides of the lumbar safety triangle progressively increased from the L2–L3 level to the L4–L5 level, in the coronal and coronal oblique planes. The overall mean triangle height was progressively greater than the base, which corroborates data in the literature^(32)^. However, one study described a lumbar safety triangle in which the base was greater than the height^(6)^. Vialle et al.^(33)^ presented data that are comparable to ours, including descriptions of the dimensions of the triangle and the spatial relationship between the dorsal root ganglion and the triangle. In the coronal oblique plane, despite the progressive increase in the dimensions of the triangle across the levels, those dimensions were smaller than those obtained in the coronal plane. To our knowledge, Pairaiturkar et al.^(22)^ are the only other authors who have described and analyzed the lumbar safety triangle in the coronal oblique plane. Those authors studied sagittal, axial, and oblique MRI measurements of the lumbar spine in 50 patients. In their study, the maximum endoscopic cannula diameter of 4–8 mm was adequate for most (62%) of the patients studied, with only 2% receiving a cannula larger than 8 mm. However, the authors did not mention the area of the triangle or the dorsal root ganglion.

We found that the area of the lumbar safety triangle increased progressively from the L2–L3 level to the L4–L5 level, which is in agreement with the findings of cadaver studies conducted by Hoshide et al.^(34)^ and Kumari et al.^(35)^, who found no statistical difference between the left and right sides in terms of the area of the triangle. However, in another cadaver study, Hardenbrook et al.^(36)^ demonstrated that the area of the safe zone for accessing the disc space can vary according to the level, including a comparison of the measurements of the areas of two possible safe zones.

Lertudomphonwanit et al.^(37)^ proposed a new safe zone shape. The authors stated that the format that would best define this corridor would be a trapezoidal shape, the area varying according to the level studied, and that it could even be increased by distracting the disc space or by manipulating the nerve root. However, they did not mention the nerve root ganglion.

One of the strengths of our study is the analysis of the dorsal root ganglion and its relationship with the corridor of safe access to the disc. In a study analyzing the root ganglion and its relationship with the safe corridor, Vialle et al.^(33)^ proposed a rectangular zone of safe access to the disc. Dannebrock et al.^(12)^ also highlighted the importance of analyzing the relationship between the dorsal root ganglion and the lumbar safety triangle, as evidenced by the fact that invasion of the triangle by the ganglion was identified on all of the images analyzed.

While many authors have compared the area and shapes of the safe zone for accessing the disc space, some studies have compared the lumbar safety triangle measurements obtained with the patient in different positions. One anatomical study, involving the analysis of 1.5-T MRI scans acquired with patients in the prone and lateral positions, showed that the measurements of the lumbar safety triangle were greater in the lateral position^(38)^. Because our study was retrospective, all of the MRI scans were acquired with the patients in the same (supine) position. Nevertheless, all of the examinations were performed in 3.0-T scanners, which has been shown to improve the accuracy of the imaging and analysis^(39)^.

When analyzing the results obtained for the lumbar safety triangle in men and women, we found that the individual aspects and overall area of the triangle were always smaller in the women than in the men. The pattern of a gradual increase in area from L2 to L5 was seen in both sexes. There was also an increase in the dimensions of the area of the lumbar safety triangle from the first age group (18–9 years) to the second (40–5 years), with lower values being observed in the patients over 65 years of age, in the coronal and coronal oblique planes. One possible explanation for these findings is the fact that degenerative changes resulting from normal aging after 65 years of age lead to changes in the configuration and area of the triangle^(40)^. To our knowledge, this is the first study to compare the dimensions of the lumbar safety triangle by sex and age group.

With the advent of modern techniques and high-definition imaging, the dorsal root ganglion has been studied in greater detail^(17)^. Most ganglia are located directly below the vertebral pedicles, with one third overlapping a lateral portion of the intervertebral disc^(41)^. The present study showed a progressive increase in the dimensions of the ganglion from the L2 level to the L5 level, those dimensions being greater in the coronal plane. Our findings corroborate those of previous studies describing a gradual increase^(13,14)^, including larger dimensions in men^(13)^, which is also in agreement with our findings, in the coronal and coronal oblique planes. The dorsal root ganglion can be further classified, according to its anatomical position, as intraspinal, intraforaminal, or extraforaminal. The L4 and L5 roots are typically intraforaminal, whereas the S1 root is typically intraspinal^(42)^. That was one of the reasons why the L5–S1 level was not investigated in our study.

In all of the images evaluated in our study, the dorsal root ganglion was found to be invading the lumbar safety triangle, as described in previous studies^(12,33)^. In general, the highest degree of invasion, at all levels, was seen in the coronal plane, with a progressive increase from the L2 level to the L5 level. That can be explained by the fact that the area of the triangle was also larger in the coronal plane than in the coronal oblique plane.

Because the lumbar safety triangle is a three-dimensional structure, the advent of new techniques and image reconstructions in different dimensions has made the study of the dimensions of the triangle in different views and planes important in the preoperative planning of minimally invasive procedures^(43)^. In our comparative study of measurements of the lumbar safety triangle in the coronal and coronal oblique planes, a gradual increase in the measurements from the L2 level to the L5 level was seen at all levels and in both planes. We also found that, at all levels, the boundaries of the lumbar safety triangle were smaller in the coronal oblique plane than in the coronal plane, with the mean differences between the two planes being statistically significant for almost all measures. Recent studies have performed three-dimensional computed tomography^(44)^ and MRI reconstructions in preoperative planning software^(15,45)^, suggesting the appropriate location and safe angulation for introducing the working cannulas^(46)^. However, those studies have not mentioned the measurements of the lumbar safety triangle at those angulations.

The strength of our study is that we analyzed the lumbar safety triangle in two planes, as well as that we measured all of the boundaries of the triangle and that we included the dimensions of the dorsal root ganglion. However, the analysis of a three-dimensional structure in only two planes is a potential limitation of our study, which could somehow interfere with the results obtained. Another potential limitation is that the images were not analyzed by independent observers. There is a need for further studies to compare different planes, such as the coronal, axial, and sagittal oblique planes, for the preoperative analysis of patients who will undergo minimally invasive procedures involving the lumbar spine.

## CONCLUSION

The dimensions and area of the lumbar safety triangle progressively increased from the L2–L3 level to the L3–L4 and L4–L5 levels, in the coronal and coronal oblique planes. The area and the boundaries of the lumbar safety triangle were both significantly smaller in the coronal oblique plane than in the coronal plane. The dorsal root ganglion invaded the triangle at all levels. Evaluation of the lumbar spine by MRI in the coronal oblique plane can increase the safety of percutaneous procedures in the triangle by revealing its exact position, as well as the degree of the dorsal root invasion into this three-dimensional structure.

## References

[1] Gautschi OP, Stienen MN, Corniola MV (2014). Minimal-invasive lumbale
Wírbelsäulenchírurgíe: historischer
Rückblick, aktueller Stand und Ausblick. Praxis.

[2] Oppenheimer JH, DeCastro I, McDonnell DE (2009). Minimally invasive spine technology and minimally invasive spine
surgery: a historical review. Neurosurg Focus.

[3] Kim DY, Lee SH, Chung SK (2005). Comparison of multifidus muscle atrophy and trunk extension
muscle strength: percutaneous versus open pedicle screw
fixation. Spine (Phila Pa 1976).

[4] Mayer HM, Brock M (1993). Percutaneous endoscopic discectomy: surgical technique and
preliminary results compared to microsurgical discectomy. J Neurosurg.

[5] Kambin P, Gellman H (1983). Percutaneous lateral discectomy of the lumbar spine: a
preliminary report. Clin Orthop Relat Res.

[6] Mirkovic SR, Schwartz DG, Glazier KD (1995). Anatomic considerations in lumbar posterolateral percutaneous
procedures. Spine (Phila Pa 1976).

[7] Quester R, Schroder R (1997). The shrinkage of the human brain stem during formalin fixation
and embedding in paraffin. J Neurosci Methods.

[8] Huang X, Zhu B, Liu X (2018). Quantitative 3D trajectory measurement for percutaneous
endoscopic lumbar discectomy. Pain Physician.

[9] Krishna M, Pollock RD, Bhatia C (2008). Incidence, etiology, classification, and management of neuralgia
after posterior lumbar interbody fusion surgery in 226
patients. Spine J.

[10] Chhabra A, Zhao L, Carrino JA (2013). MR neurography: advances. Radiol Res Pract.

[11] Chhabra A, Lee PP, Bizzell C (2011). 3 Tesla MR neurography–technique, interpretation, and
pitfalls. Skeletal Radiol.

[12] Dannebrock FA, Zardo EA, Ziegler MS (2019). Evaluation of the lumbar safety triangle through magnetic
resonance imaging. Coluna/Columna.

[13] Shen J, Wang HY, Chen JY (2006). Morphologic analysis of normal human lumbar dorsal root ganglion
by 3D MR imaging. AJNR Am J Neuroradiol.

[14] Hasegawa T, Mikawa Y, Watanabe R (1996). Morphometric analysis of the lumbosacral nerve roots and dorsal
root ganglia by magnetic resonance imaging. Spine (Phila Pa 1976).

[15] Hirayama J, Hashimoto M, Sakamoto T (2020). Clinical outcomes based on preoperative Kambin’s triangular
working zone measurements on 3D CT/MR fusion imaging to determine optimal
approaches to transforaminal endoscopic lumbar diskectomy. J Neurol Surg A Cent Eur Neurosurg.

[16] Min JH, Kang SH, Lee JB (2005). Morphometric analysis of the working zone for endoscopic lumbar
discectomy. J Spinal Disord Tech.

[17] Ohmori K, Kanamori M, Kawaguchi Y (2001). Clinical features of extraforaminal lumbar disc herniation based
on the radiographic location of the dorsal root ganglion. Spine (Phila Pa 1976).

[18] Wiltse LL (2000). Anatomy of the extradural compartments of the lumbar spinal
canal. Peridural membrane and circumneural sheath. Radiol Clin North Am.

[19] Puigdellívol-Sánchez A, Prats-Galino A, Ruano-Gil D (1998). Sciatic and femoral nerve sensory neurones occupy different
regions of the L4 dorsal root ganglion in the adult rat. Neurosci Lett.

[20] Kobayashi S, Yoshizawa H, Yamada S (2004). Pathology of lumbar nerve root compression. Part 2: morphological
and immunohistochemical changes of dorsal root ganglion. J Orthop Res.

[21] Guan X, Gu X, Zhang L (2015). Morphometric analysis of the working zone for posterolateral
endoscopic lumbar discectomy based on magnetic resonance
neurography. J Spinal Disord Tech.

[22] Pairaiturkar PP, Sudame OS, Pophale CS (2019). Evaluation of dimensions of Kambin’s triangle to calculate
maximum permissible cannula diameter for percutaneous endoscopic lumbar
discectomy: a 3-dimensional magnetic resonance imaging based
study. J Korean Neurosurg Soc.

[23] Cohen J (1992). A power primer. Psychol Bull.

[24] Choi G, Kang HY, Modi HN (2011). Risk of developing seizure after percutaneous endoscopic lumbar
discectomy. J Spinal Disord Tech.

[25] Choi I, Ahn JO, So WS (2013). Exiting root injury in transforaminal endoscopic discectomy:
preoperative image considerations for safety. Eur Spine J.

[26] Hsu HT, Chang SJ, Yang SS (2013). Learning curve of full-endoscopic lumbar
discectomy. Eur Spine J.

[27] Yeung AT, Tsou PM (2002). Posterolateral endoscopic excision for lumbar disc herniation:
surgical technique, outcome, and complications in 307 consecutive
cases. Spine (Phila Pa 1976).

[28] Wang H, Zhou Y, Zhang Z (2016). Postoperative dysesthesia in minimally invasive transforaminal
lumbar interbody fusion: a report of five cases. Eur Spine J.

[29] Tumialán LM, Madhavan K, Godzik J (2019). The history of and controversy over Kambin’s triangle: a
historical analysis of the lumbar transforaminal corridor for endoscopic and
surgical approaches. World Neurosurg.

[30] Park KD, Lee J, Jee H (2012). Kambin triangle versus the supraneural approach for the treatment
of lumbar radicular pain. Am J Phys Med Rehabil.

[31] Ozer AF, Suzer T, Can H (2017). Anatomic assessment of variations in Kambin’s triangle: a
surgical and cadaver study. World Neurosurg.

[32] Choi PS, Basile Júnior R (2003). Estudo anatômico da zona triangular de segurança
aplicada aos procedimentos percutâneos
póstero-laterais. Coluna/Columna.

[33] Vialle E, Vialle LR, Contreras W (2015). Anatomical study on the relationship between the dorsal root
ganglion and the intervertebral disc in the lumbar spine. Rev Bras Ortop.

[34] Hoshide R, Feldman E, Taylo W (2016). Cadaveric analysis of the Kambin’s triangle. Cureus.

[35] Kumari C, Gupta T, Gupta R (2021). Cadaveric anatomy of the lumbar triangular safe zone of Kambin’s
in North West Indian population. Anat Cell Biol.

[36] Hardenbrook M, Lombardo S, Wilson MC (2016). The anatomic rationale for transforaminal endoscopic interbody
fusion: a cadaveric analysis. Neurosurg Focus.

[37] Lertudomphonwanit T, Keorochana G, Kraiwattanapong C (2016). Anatomic considerations of intervertebral disc perspective in
lumbar posterolateral approach via Kambin’s triangle: cadaveric
study. Asian Spine J.

[38] Botanlioğlu H, Aydingöz Ö, Kantarci F (2015). Positional alterations of the Kambin’s triangle and foraminal
areas in the lumbosacral region. Acta Orthop Traumatol Turc.

[39] Ensle F, Kaniewska M, Tiessen A (2023). Diagnostic performance of deep learning-based reconstruction
algorithm in 3D MR neurography. Skeletal Radiol.

[40] Choma TJ, Rechtine GR, McGuire Jr RA (2015). Treating the aging spine. J Am Acad Orthop Surg.

[41] Moon HS, Kim YD, Song BH (2010). Position of dorsal root ganglia in the lumbosacral region in
patients with radiculopathy. Korean J Anesthesiol.

[42] Kikuchi S, Sato K, Konno S (1994). Anatomic and radiographic study of dorsal root
ganglia. Spine (Phila Pa 1976).

[43] Fan G, Liu H, Wu Z (2019). Deep learning-based automatic segmentation of lumbosacral nerves
on CT for spinal intervention: a translational study. AJNR Am J Neuroradiol.

[44] Yu P, Wang Y, Wu X (2020). A digital anatomic investigation of the safe triangle areas for
L1-5 percutaneous minimally invasive discectomy. Surg Radiol Anat.

[45] Chen X, Cheng J, Gu X (2016). Development of preoperative planning software for transforaminal
endoscopic surgery and the guidance for clinical
applications. Int J Comput Assist Radiol Surg.

[46] Fan G, Guan X, Zhang H (2015). Significant improvement of puncture accuracy and fluoroscopy
reduction in percutaneous transforaminal endoscopic discectomy with novel
lumbar location system: preliminary report of prospective HELLO
study. Medicine (Baltimore).

